# Affiliate Stigma among family caregivers of individuals with dementia in China: a cross-sectional study

**DOI:** 10.3389/fpubh.2024.1366143

**Published:** 2024-05-30

**Authors:** Yingying Shi, Shishi Dong, Zhiqi Liang, Mengting Xie, Hanyi Zhang, Sixie Li, Jufang Li

**Affiliations:** ^1^School of Nursing, Wenzhou Medical University, Zhejiang, China; ^2^Department of Neurology, Sir Run Run Shaw Hospital, Zhejiang University School of Medicine, Zhejiang, China

**Keywords:** dementia, family caregiver, affiliate stigma, China, caregiver burden

## Abstract

**Background:**

Affiliate stigma experienced by family caregivers of individuals with dementia may seriously affect home care and prognosis of these patients. This study aimed to explore the levels of perceived affiliate stigma and its influencing factors among family caregivers of patients with dementia in mainland China, which remains a relatively unexplored topic.

**Methods:**

In this cross-sectional study, purposive sampling was used to recruit dementia family caregivers from an online communication group between April and May 2022. A total of 727 eligible caregivers were included and asked to complete the demographic questionnaire, the affiliate stigma scale, and the caregiver burden inventory. Descriptive statistics, independent sample t-test, one-way analysis of variance, Pearson correlation analysis, and multiple linear regression were used to explore the factors that influence perceived affiliate stigma among dementia family caregivers.

**Results:**

The mean score for affiliate stigma of dementia family caregivers was 48.09 ± 16.38 (range: 22–86). Whether there were regular breaks during patient care, time-dependent burden, developmental burden, physical burden, and social burden were significant factors influencing the affiliate stigma of dementia family caregivers.

**Conclusion:**

Dementia family caregivers showed a moderate to high level of affiliate stigma. Those who had regular breaks during patient care, higher time-dependent burden, developmental burden, and physical burden and lower social burden exhibited higher levels of affiliate stigma.

## 1 Introduction

Dementia is a highly prevalent disease in people older than 65 years and has been diagnosed in increasingly younger individuals in recent years (1–3). There are currently approximately 600,000 people with dementia in China, which means that 7 out of every 100 people older than 65 years have dementia (4). As dementia progresses, patients gradually lose the ability to perform daily activities and require the assistance of healthcare professionals and caregivers. Thus, dementia is recognized as a huge social and healthcare challenge in China (5). With the advancement of the universal medical insurance system in China, the financial burden of treatment and care faced by patients with dementia has eased (5). However, the medical insurance coverage for long-term care is limited, and most families in China cannot afford expensive commercial care services. As a result, patients with dementia still rely heavily on informal home care (5). It is estimated that 75% of patients with dementia are predominantly cared for by family caregivers (6, 7). Owing to the lack of well-developed family support services in China (5), most family caregivers have limited knowledge and skills in caring for patients with dementia (5, 8). As a result, family caregivers suffer from increased negative emotions when caring for patients with dementia who exhibit symptoms of delusions, hallucinations, and aggressive behaviors (9–11). Specifically, a meta-analysis reported a high prevalence of depression (34.0%) and anxiety (43.6%) among dementia family caregivers (12), and they were particularly vulnerable to affiliate stigma (11, 13–15).

Affiliate stigma is defined as the process by which the stigmatized individual internalizes the negative reactions of others to him or her (16). Affiliate stigma has a further negative impact on family caregivers’ physical and mental health (17). In addition, affiliate stigma increases social withdrawal in dementia family caregivers and is an important cause of negative emotions (11, 15). Specifically, family caregivers with affiliate stigma experience high level of stress, high caregiver burden, and engage less in help-seeking behaviors, which, in turn, affects the quality of care they provide to patients with dementia (18–20). Moreover, researchers conducting in-depth interviews with child caregivers found that they experienced feelings of shame and disgust due to the caring activity while feeling guilt and self-blame for their shame and disgust toward their parents (19). These perceptions contribute to a growing sense of stigma among dementia family caregivers, which must be urgently alleviated (19). Although the mental health needs of dementia family caregivers are gaining widespread attention, research examining affiliate stigma in this context is still in its infancy. Understanding the levels of affiliate stigma among dementia family caregivers and the influencing factors involved is necessary for healthcare professionals to develop effective strategies to reduce affiliate stigma in this group. To address this gap, two research questions were proposed: (1) What is the level of affiliate stigma among dementia family caregivers in China? (2) What are the factors that influence affiliate stigma among dementia family caregivers in China?

## 2 Background

### 2.1 Levels of affiliate stigma of dementia family caregivers

Affiliate stigma has been reported to occur among dementia family caregivers in many regions, such as Iran, China, Vietnam, Singapore, Ethiopia, the United Kingdom, Belgium, Taiwan, Israel, America, Hong Kong, Malaysia, South Korea, Brazil, Nigeria, Indonesia, and Ghana (16, 21–36). Although the specific rates of incidence have rarely been reported, some studies have indicated the high incidence of affiliate stigma among dementia family caregivers. According to a study from Israel, 50% of a sample of dementia family caregivers experienced affiliate stigma as a result of caring for a relative with dementia-like conditions (28). Furthermore, a qualitative study reported that 91% of Asian-American dementia family caregivers mentioned about stigma (21). In addition, stigmatization of primary caregivers for individuals with mental illness was reported to be 94 and 75% in studies from Singapore (22) and Ethiopia (23), respectively. Moderate to high levels of affiliate stigma have been reported among dementia family caregivers in mainland China (27), while the affiliate stigma levels of dementia family caregivers in Iran are found to be even higher (16). However, dementia family caregivers’ affiliate stigma levels were lower in Taiwan, United Kingdom, and Belgium (24–26).

### 2.2 Influencing factors of dementia family caregivers’ affiliate stigma

Levels of affiliate stigma among dementia family caregivers were found to be influenced by demographic characteristics (such as caregivers’ gender, age, place of residence, relationship with patient, and patients’ gender) and psychological characteristics (such as anxiety and depression). Specifically, female caregivers experienced more affiliate stigma than male caregivers (37), although another study reported the opposite result (14). Younger caregiver age was associated with higher affiliate stigma (38). Caregivers living in rural areas showed higher scores for affiliate stigma than caregivers living in urban areas (39). Children caregivers experienced higher affiliate stigma than spouse caregivers (37). In addition, male patients with dementia are more aggressive and disruptive, exacerbating the stigma perceived by female spouse caregivers (40). In addition to these demographic variables, anxiety was significantly associated with affiliate stigma (14), while depression predicted affiliate stigma either directly (13) or indirectly through caregiver burden (41). Furthermore, a qualitative study of dementia family caregivers in the Asian-American population found that stigma is strongly associated with negative stereotypes of older adult care and progression of chronic disease (21).

Current research on the factors influencing dementia family caregivers’ affiliate stigma has mainly focused on demographic variables, and few psychological factors have been explored. In this study, the dimensions of caregiver burden were additionally considered as main variables that may influence dementia family caregivers’ affiliate stigma. Caregiver burden refers to caregivers’ multifaceted stress levels arising from caring for a relative and includes time-dependent burden, developmental burden, physical burden, social burden, and emotional burden (3, 42). Caregivers with the same total burden score may experience different patterns of burden and therefore require differing interventions (43). In addition, different burden dimensions may have varying degrees of influence on affiliate stigma. Therefore, we attempted to explore the impact of caregiver burden on affiliate stigma from the perspective of the various dimensions of this burden. Caregiver burden was confirmed to be positively related to affiliate stigma, and both variables were positively correlated with all dimensions (13, 14, 37). Specifically, caregivers with higher caregiver burden experienced higher affiliate stigma and were unable to provide high-quality care (14). Furthermore, highly burdened caregivers may experience more public stigma, which may lead to high levels of affiliate stigma (44). Furthermore, another study (26) suggested that dementia caregivers caring for a family member with disruptive behavior may experience embarrassment and shame. This could be regarded as emotional burden, which was also associated with affiliate stigma. Although the relationship between caregiver burden and affiliate stigma has been addressed by some researchers, few studies focus on its impact on affiliate stigma from the perspective of the dimensions of caregiver burden.

In summary, current research on affiliate stigma levels is limited. In addition, extant studies report inconsistent findings in regard to the factors that influence affiliate stigma. Furthermore, perceived affiliate stigma levels and their influencing factors among dementia family caregivers in mainland China remain a relatively unexplored topic, especially from the perspective of burden dimensions. Hence, the purpose of this study was to explore the levels of affiliate stigma and its influencing factors from the perspective of burden dimensions among dementia family caregivers in mainland China.

## 3 Methods

### 3.1 Study design

This was a cross-sectional study conducted to examine the level of affiliate stigma and its influencing factors among dementia family caregivers in mainland China.

### 3.2 Setting and sample

Purposive sampling was used to recruit dementia family caregivers from April to May 2022. In purposive sampling, the characteristics of the sample are defined for a purpose that is relevant to the study. The rationale of using purposive sampling is as follows (45): First, purposive sampling avoids random forms of sampling and ensures that the specific types of cases included are part of the final sample for the study. Second, purposive sampling enhances transferability by identifying study participants in terms of inclusion and exclusion criteria, which helps determine the applicability of study findings to other situations and populations. In this study, specific inclusion and exclusion criteria were defined to recruit dementia family caregivers from an online communication group. All members in the online communication group were dementia family caregivers from mainland China who were eligible to represent the target population. For inclusion in this study, caregivers were required to meet the following criteria: (1) primary caregivers of the dementia patient; (2) aged 20 years or older; (3) able to understand the purpose of the study and the content of the study questionnaires; (4) only cared for the dementia patient, and (5) cared for the dementia patient for more than 3 months. The exclusion criterion was receipt of money from caregiving activities. The sample size was determined by combining the following two methods: (1) Power analysis was conducted using G*Power 3.1.9.2 software (46, 47), with an effect size of 0.15, a significance level of 0.05, and a power of 0.80. The sample was calculated to be 117 cases after setting a sample loss rate of 20%. (2) Rough sample size estimation method was adopted, which required the sample size to be 10 to 20 times of the number of study variables (48). In total, 32 variables were included in this study, and the sample size was calculated to be at least 320 to 640 cases. Taking into account invalid questionnaires, the sample size was further expanded by 20% as 384 to 768 cases. In summary, to obtain more robust statistical results, we attempted to collect as much data as possible and set the sample size to at least 384 cases. Finally, 1,024 questionnaires were collected, out of which 727 questionnaires were valid. The effective response rate was 71.0%.

### 3.3 Measures

#### 3.3.1 The demographic questionnaire

The demographic questionnaire was designed by the researchers based on a literature review (22, 37, 49–51), to collect information on the dementia family caregivers’ gender, age, ethnic group, religious beliefs, educational level, marital status, employment status, whether regular breaks were taken during patient care, monthly household income, relationship with the patient, whether the carer lived with the patient, whether the carer received assistance in caring for the patient, average time spent caring for the patient per day, and knowledge of dementia. In addition, questions regarding the patients’ gender, age, ethnic groups, religious beliefs, marital status, type of medical insurance, course of disease, and self-care ability were included in the demographic questionnaire.

#### 3.3.2 Affiliate stigma scale

The affiliate stigma scale, which was developed to assess affiliate stigma levels of dementia caregivers by Mak and Cheung (52) in Cantonese, comprises 22 items with three dimensions, namely, affective, behavioral, and cognitive. All items are rated on a four-point Likert scale ranging from 1 (not at all) to 4 (always). The total score ranged from 22 to 88, with higher scores indicating higher levels of affiliate stigma. The affiliate stigma scale was reliable, as evidenced by Cronbach’s α of 0.94 (52). The Cantonese version of the affiliate stigma scale was converted into the Mandarin version by the research team, without any modification, as requested by the original author. The Mandarin version was used in this study. In this study, Cronbach’s α of total affiliate stigma scale was 0.970, and Cronbach’s α for the dimensions of affective, behavioral, and cognitive dimensions were 0.901, 0.927, and 0.927, respectively.

#### 3.3.3 Caregiver burden inventory

The caregiver burden inventory, which was developed by Novak and Guest (43) and was translated into Chinese by Yue (53), was used to assess dementia family caregivers’ burden. The caregiver burden inventory consists of 24 items with five dimensions, namely, emotional burden, social burden, time-dependent burden, developmental burden, and physical burden. All items are rated on a five-point Likert scale ranging from 0 (not at all) to 4 (always). The total score ranges from 0 to 96, with higher scores indicating higher levels of caregiver burden. Cronbach’s α of total caregiver burden inventory was 0.92, and the range of each dimension was 0.68 to 0.93 (time-dependent burden = 0.93, developmental burden = 0.83, physical burden = 0.83; social burden = 0.68; and emotional burden = 0.78) (53). In this study, Cronbach’s α of total caregiver burden inventory was 0.958, and the range of each dimension was 0.852 to 0.890 (time-dependent burden = 0.890, developmental burden = 0.879, physical burden = 0.852, social burden = 0.849, and emotional burden = 0.889).

### 3.4 Data collection

Data were collected using online electronic questionnaires, with the consent of the administrator of a dementia family caregiver online communication group. The questionnaires were entered into China’s largest online survey platform to generate a quick response code for distributing the questionnaires. Then, the quick response code was sent to the dementia family caregiver online communication group. Potential participants scanned the quick response code with their mobile phones and responded to the questionnaires online. To ensure that all questions were answered, the online survey rules were set to refuse the submission of a survey if any questions had not been answered. Questionnaires could be submitted from a single internet protocol address only once.

### 3.5 Data analysis

SPSS 26.0 was used to analyze the data. Categorical variables were described as frequencies and percentages. Numerical variables were described by means and standard deviations. Independent sample t-test and one-way analysis of variance were used to investigate the differences in dementia family caregivers’ affiliate stigma based on the family caregivers’ and patients’ demographic characteristics. Pearson correlation analysis was used to detect the correlation between dementia family caregivers’ burden and affiliate stigma and the relationship between the numerical demographic variables and affiliate stigma. Multiple linear regression was used to explore the factors influencing dementia family caregivers’ affiliate stigma. Nominal variables were converted into dummy variables before entering the regression model. A difference of *p* < 0.05 was considered to indicate statistical significance.

## 4 Results

### 4.1 Demographic characteristics of dementia family caregivers and patients with dementia

The demographic characteristics of the family caregivers and patients with dementia are shown in Table 1. The average age of the family caregivers was 39.12 years (standard deviation = 9.67, range: 21–77), and their average time spent caring for patients per day was 5.44 h (standard deviation = 3.01, range: 1–15). Slightly more than half of the family caregivers were women (50.5%). More than two-fifths of the family caregivers had an undergraduate educational level (40.3%) and a monthly household income of more than 5,001 RMB (41.1%). More than one-third of the family caregivers had a basic knowledge of dementia (35.5%). The majority of the family caregivers were of Han ethnicity (99.6%), with no religious beliefs (73.5%), married (85.1%), employed (84.0%), children of the patients with dementia (78.4%), took regular breaks during patient care (85.0%), living with the patients with dementia (87.2%), and received assistance in caring for the patients with dementia (91.5%). The average age of the patients with dementia was 65.21 years (standard deviation =8.17, range: 52–92), and the average course of disease was 5.56 years (standard deviation =3.29, range: 1–20). The majority of the patients were male (54.9%) of Han ethnicity (99.6%), with no religious beliefs (70.4%), married (72.2%), having new rural cooperative medical insurance (51.3%), and had partial self-care abilities (76.3%).

**Table 1:** Demographic characteristics and univariate analysis of affiliated stigma among family caregivers and patients with dementia (*N* = 727).

Variables	*N* (%)	Mean ± standard deviations	t/F/r	P
**Family caregivers**				
Gender			2.412	0.016
Male	360 (49.5)	49.57 ± 16.78		
Female	367 (50.5)	46.65 ± 15.87		
Ethnic group			0.514	0.656
Han nationality^1^	724 (99.6)	48.10 ± 16.40		
Other	3 (0.4)	46.00 ± 7.00		
Religious beliefs			2.697	0.007
Yes	193 (26.5)	50.93 ± 17.50		
No	534 (73.5)	47.07 ± 15.84		
Educational level			2.107	0.063
Primary school or below	7 (1.0)	59.14 ± 17.48		
Junior high school	52 (7.2)	48.12 ± 16.97		
High school or technical secondary school	188 (25.9)	50.42 ± 16.91		
Junior college	180 (24.8)	48.14 ± 17.13		
Undergraduate	293 (40.3)	46.37 ± 15.38		
Masters or above	7 (1.0)	45.29 ± 7.37		
Marital status			7.041	<0.001
Had a spouse	619 (85.1)	47.26 ± 16.36		
Unmarried	70 (9.6)	49.26 ± 16.89		
Divorced	27 (3.7)	59.85 ± 10.17		
Widowed	11 (1.5)	58.82 ± 11.65		
State of work			1.648	0.193
On the job	611 (84.0)	47.62 ± 16.39		
Out of work	86 (11.8)	50.83 ± 13.89		
Retirement	30 (4.1)	49.93 ± 16.38		
Whether there were regular breaks during patient care			2.369	0.019
Yes	618 (85.0)	48.65 ± 16.56		
No	109 (15.0)	44.94 ± 14.75		
Monthly household income (RMB)			2.927	0.033
Less than 3,000	11 (1.5)	57.73 ± 11.54		
3,001–5,000	149 (20.5)	49.97 ± 16.18		
5,001–7,000	299 (41.1)	48.33 ± 17.04		
More than 7,000	268 (36.9)	46.39 ± 15.70		
Relationship with patient			5.907	0.003
Spouse	50 (6.9)	54.62 ± 19.66		
Children	570 (78.4)	47.13 ± 16.01		
Relatives	107 (14.7)	50.18 ± 15.91		
Whether lived with the patient			1.238	0.216
Yes	634 (87.2)	48.38 ± 16.47		
No	93 (12.8)	46.13 ± 15.70		
Whether had assistance in caring for the patient			−0.367	0.714
Yes	665 (91.5)	48.02 ± 16.47		
No	62 (8.5)	48.02 ± 15.45		
Knowledge of dementia			11.009	<0.001
Completely lacking knowledge	12 (1.7)	64.92 ± 12.93		
A small amount of knowledge (knowing a small amount about dementia)	221 (30.4)	51.04 ± 16.73		
Basic knowledge (having basic knowledge and nursing skills in relation to dementia)	258 (35.5)	49.21 ± 15.95		
Most knowledge (having a high level of knowledge and nursing skills in relation to dementia)	171 (23.5)	43.41 ± 14.38		
Full knowledge	65 (8.9)	42.83 ± 17.49		
Age of the family caregivers		39.12 ± 9.67	−0.009	>0.05

Average time spent caring for patients per day (hour)		5.44 ± 3.01	0.055	>0.05
**Patients with dementia**				
Gender			−1.749	0.081
Male	399 (54.9)	47.13 ± 16.19		
Female	328 (45.1)	49.26 ± 16.55		
Ethnic groups			0.222	0.825
Han nationality^1^	724 (99.6)	48.10 ± 16.41		
Other	3 (0.4)	46.00 ± 7.00		
Religious beliefs			4.301	<0.001
Yes	215 (29.6)	52.27 ± 17.54		
No	512 (70.4)	46.34 ± 15.56		
Marital status			6.942	<0.001
Had a spouse	525 (72.2)	47.25 ± 16.48		
Unmarried	11 (1.5)	63.36 ± 11.46		
Divorced	23 (3.2)	58.52 ± 14.03		
Widowed	168 (23.1)	48.31 ± 15.73		
Types of medical insurance			4.014	0.003
Urban employee basic medical insurance	196 (27.0)	44.68 ± 15.26		
New rural cooperative medical insurance	373 (51.3)	49.26 ± 17.18		
Urban residents basic medical insurance	150 (20.6)	49.04 ± 15.14		
Commercial medical insurance	6 (0.8)	56.17 ± 13.63		
No insurance	2 (0.2)	69.5 ± 12.02		
Self-care ability			11.53	<0.001
Fully capable of self-care (could independently complete the activities of daily living, such as bathing, dressing, toileting, moving, bowel and bladder control, and eating)	99 (13.6)	53.86 ± 18.90		
Partially capable of self-care (able to perform some activities of daily living independently, such as bathing, dressing, toileting, moving, bowel and bladder control, and eating but requiring assistance from others)	555 (76.3)	46.50 ± 15.40		
Completely unable to take care of themselves (could not independently perform bathing, dressing, toileting, moving, bowel and bladder control, and eating; all requiring assistance from others)	73 (10.0)	52.34 ± 17.58		
Age of the patients (years)		65.21 ± 8.17	0.033	>0.05

The course of disease (years)		5.56 ± 3.30	0.027	>0.05

^1^The largest ethnic group in China with a proportion of appoximately 91.59%.

### 4.2 Level of dementia family caregivers’ affiliate stigma and burden

The mean scores of total affiliate stigma and each dimension are presented in Table 2. The total affiliate stigma score of dementia family caregivers was 48.09 (standard deviation = 16.38), which corresponded to a moderate to high level. The mean scores for the affective, behavioral, and cognitive dimensions were 16.03 (standard deviation = 5.31), 17.25 (standard deviation = 6.18), and 14.81 (standard deviation = 5.33), respectively. In addition, the total caregiver burden score of dementia family caregivers was 47.35 (standard deviation = 21.41), which corresponded to a high level. The mean scores of its dimensions are shown in Table 2.

**Table 2:** The means and standard deviations of dementia family caregivers’ burden and affiliate stigma and the relationship between them (*N* = 727).

Variables	(Mean ± standard deviations)	X1	X2	X3	X4	X5	X6	X7	X8	X9	X10
X1: Total affiliate stigma	48.09 ± 16.38	1	–	–	–	–	–	–	–	–	–
X2: Affective dimension	16.03 ± 5.31	0.953**	1	–	–	–	–	–	–	–	–
X3: Behavioral dimension	17.25 ± 6.18	0.977**	0.898**	1	–	–	–	–	–	–	–
X4: Cognitive dimension	14.81 ± 5.33	0.964**	0.866**	0.923**	1	–	–	–	–	–	–
X5: Total burden	47.35 ± 21.41	0.808**	0.820**	0.779**	0.741**	1	–	–	–	–	–
X6: Time-dependent burden	10.31 ± 5.27	0.735**	0.761**	0.705**	0.664**	0.907**	1	–	–	–	–
X7: Developmental burden	8.07 ± 5.22	0.855**	0.820**	0.840**	0.813**	0.898**	0.786**	1	–	–	–
X8: Physical burden	6.41 ± 4.19	0.851**	0.817**	0.827**	0.817**	0.884	0.752**	0.905**	1	–	–
X9: Social burden	10.10 ± 3.97	0.339**	0.409**	0.312**	0.264**	0.684**	0.544**	0.415**	0.406**	1	–
X10: Emotional burden	12.45 ± 5.98	0.676**	0.703**	0.647**	0.609**	0.921**	0.790**	0.739**	0.740**	0.657**	1

*P < 0.05; **p < 0.01.

### 4.3 Factors influencing dementia family caregivers’ affiliate stigma

The univariate analysis showed that dementia family caregivers’ gender, religious beliefs, marital status, whether regular breaks were taken during patient care, monthly household income, relationship with patients, and knowledge of dementia were associated with their levels of affiliate stigma. Furthermore, the patients’ religious beliefs, marital status, types of medical insurance, and self-care ability were also associated with dementia family caregivers’ affiliate stigma (Table 1). The reference values of the non-continuous variables above are shown in Table 3. In addition, Pearson correlation analysis showed that the total and dimension scores of dementia family caregivers’ burden were all significantly and positively correlated with the total and dimension scores of their affiliate stigma (Table 2).

**Table 3:** The reference values for non-continuous variables.

Non-continuous variables		Reference values
Family caregivers	Gender	Male = 0; Female = 1
	Religious beliefs	Yes = 0; No = 1
	Marital status^1^	Had a spouse: Z_1_ = 0, Z_2_ = 0, Z_3_ = 0; Unmarried: Z_1_ = 1, Z_2_ = 0, Z_3_ = 0; Divorced: Z_1_ = 0, Z_2_ = 1, Z_3_ = 0; Widowed: Z_1_ = 0, Z_2_ = 0, Z_3_ = 1
	Whether there were regular breaks during patient care	Yes = 0; No = 1
	Monthly household income (RMB)	Less than 3,000 = 1; 3,001–5,000 = 2; 5,001–7,000 = 3; More than 7,000 = 4
	Relationship with patient^1^	Spouse: Z_1_ = 0, Z_2_ = 0; Children: Z_1_ = 1, Z_2_ = 0; Relatives: Z_1_ = 0, Z_2_ = 1
	Knowledge of dementia	Completely lacking knowledge =1; A small amount of knowledge (knowing something about dementia) =2; Basic knowledge (having basic knowledge and nursing skills in relation to dementia) =3; A high level of knowledge (having a good amount of knowledge and nursing skills in relation to dementia) =4; Full knowledge =5
Patients with dementia	Religious beliefs	Yes = 0; No = 1
	Marital status^1^	Had a spouse: Z_1_ = 0, Z_2_ = 0, Z_3_ = 0; Unmarried: Z_1_ = 1, Z_2_ = 0, Z_3_ = 0; Divorced: Z_1_ = 0, Z_2_ = 1, Z_3_ = 0; Widowed: Z_1_ = 0, Z_2_ = 0, Z_3_ = 1
	Medical insurance type^1^	Urban employee basic medical insurance: Z_1_ = 0, Z_2_ = 0, Z_3_ = 0, Z_4_ = 0; New rural cooperative medical insurance: Z_1_ = 1, Z_2_ = 0, Z_3_ = 0, Z_4_ = 0; Urban residents basic medical insurance: Z_1_ = 0, Z_2_ = 1, Z_3_ = 0, Z_4_ = 0; Commercial medical insurance: Z_1_ = 0, Z_2_ = 0, Z_3_ = 1, Z_4_ = 0; No insurance: Z_1_ = 0, Z_2_ = 0, Z_3_ = 0, Z_4_ = 1
	Self-care ability	Fully capable of self-care (could independently complete activities of daily living, such as bathing, dressing, toileting, moving, bowel and bladder control, eating, etc.) = 1; Partially capable of self-care (able to perform some activities of daily living independently, such as bathing, dressing, toileting, moving, bowel and bladder control, and eating, etc., but requiring assistance from others) = 2; Completely unable to take care of themselves (could not complete bathing, dressing, toileting, moving, bowel and bladder control, eating, etc.; all requiring assistance from others) =3

^1^Dummy variables were set.

Factors associated with dementia family caregivers’ affiliate stigma in the univariate analysis and Pearson correlation analysis were included in the multiple linear regression model. The results showed that time-dependent burden, developmental burden, physical burden, social burden, and whether regular breaks were taken during patient care influenced dementia family caregivers’ affiliate stigma. In descending order of their magnitude of influence, the five variables were developmental burden (*β* = 0.393), physical burden (*β* = 0.392), time-dependent burden (*β* = 0.176), social burden (*β* = −0.080) and whether regular breaks were taken during patient care (yes) (*β* = −0.036). See Table 4 for details.

**Table 4:** Multiple linear regression analysis of dementia family caregivers’ affiliate stigma (*N* = 727).

Variable	Unstandardized regression coefficient		Standardized regression coefficient (*β*)	*P*	95% CI for *B*		Statistics of collinearity	
	*B*	Standard error			Lower limit	Upper limit	Tolerance of tolerance	VIF
Constant	27.909	1.256		<0.001	25.444	30.374		
Time-dependent burden (original value)	0.545	0.098	0.176	<0.001	0.352	0.738	0.312	3.202
Developmental burden (original value)	1.234	0.141	0.393	<0.001	0.957	1.511	0.155	6.457
Physical burden (original value)	1.529	0.165	0.392	<0.001	1.205	1.854	0.174	5.735
Social burden (original value)	−0.328	0.087	−0.080	<0.001	−0.499	−0.157	0.702	1.424
Whether there were regular breaks during patient care (yes = 0, no = 1)	−1.653	0.823	−0.036	0.045	−3.269	−0.037	0.970	1.031

*R* = 0.880, *R*^2^ = 0.773, *F* = 495.173, Durbin-Watson = 1.976.

## 5 Discussion

### 5.1 Levels of affiliate stigma among dementia family caregivers

The affiliate stigma scores of dementia family caregivers in China indicated that this group experienced a moderate to high level of affiliate stigma, which was higher than that reported in previous studies (24–26). This finding may be explained by the following reasons: First, a review suggested that much of the research studies on family stigma has focused on Asian countries, with Chinese culture emphasizing collectivism (54). As a result, the impact of affiliate stigma on Chinese family caregivers is obviously greater. Second, highly abnormal behaviors in people with dementia, such as agitation, irritability, defiance, wandering, cognitive impairment, and unintentional injuries (55–58), may all contribute to affiliate stigma of family caregivers. Third, the majority of the caregivers in this study were adult children of patients—a group that has previously been reported to experience high levels of affiliate stigma (37). This may also explain the higher scores of affiliate stigma observed in this study. Notably, Iranian dementia family caregivers’ level of affiliate stigma was higher than the levels found in our study (16). This may be due to the difference in the proportion of female caregivers in the samples of the two studies, i.e., two-thirds of the dementia family caregivers were women in the Iran study (16), while only half were women in this study. It is worth noting that women in both Iranian and traditional Chinese culture are expected to assume the role of caregivers. Given that researchers have found that female caregivers generally experience higher affiliate stigma (25), it is not surprising that the level of affiliate stigma was higher in the Iran study as it included more female caregivers.

### 5.2 Factors influencing dementia family caregivers’ affiliate stigma

The multiple linear regression analysis revealed that dementia family caregivers’ affiliate stigma was influenced by their time-dependent burden, developmental burden, physical burden, social burden, and whether they took regular breaks during patient care (yes).

This study found that dementia family caregivers with higher time-dependent burden exhibited higher level of affiliate stigma. Time-dependent burden is a measure of the cost of the caregivers’ time spent on caring for the patients (42). A previous study showed that time-dependent burden is associated with dementia severity (42), and the time spent by caregivers significantly increases as dementia worsens. In this case, the caregivers spend significantly more time at home, and they may even need to take the patients along with them, for the patients’ own safety, when they have to leave home. These factors reinforce the affiliated stigma of the caregivers.

Family caregivers with higher developmental burden exhibited higher affiliate stigma in our study. Developmental burden explains the sense of failure generated by caregivers compared with their peers during development (42). Dementia family caregivers may perceive themselves at a stage in life which does not match their expectations of their development at that stage. Owing to their caregiving duties, their development and progression, in terms of life goals, may have stagnated relative to their peers, which may trigger psychological imbalance. They may then strive harder to pursue their unfulfilled ambitions to meet their developmental expectations (59) and suffer more affiliate stigma in the process of socializing with their peers who are more developmentally advanced.

Our study showed that caregivers with higher physical burden reported higher affiliate stigma. Physical burden describes the caregivers’ perception of chronic fatigue and harm to physical health (42). A previous study has shown that behavioral disorders among dementia patients, such as increased nocturnal activity, incontinence, and impaired mobility, resulted in physical stress for caregivers (60), which subsequently added to their physical burden (61). In addition, with increased cognitive impairment of patients, caregivers perceive more conflict and less resilience and support in the family (62, 63). The increased physical burden and prolonged exposure to a non-supportive environment may result in incremental affiliate stigma of caregivers.

This study also found that family caregivers with higher social burden had lower levels of affiliate stigma. Social burden refers to the caregivers’ perception of role conflict (42). Higher social burden was correlated with lower affiliate stigma, which may be explained as follows: The majority of the family caregivers were the children of the patients (78.4%) who experienced higher social burden owing to a variety of social roles they performed, such as filial duties, employees, and parental duties. Multiple roles may keep them busy with their families and work, giving them little time to develop perceptions of affiliate stigma. Thus, family caregivers with higher social burden exhibited lower affiliate stigma. Conversely, family caregivers with lower social burden may not take on more social roles and focus on caring for patients with dementia, leading to higher affiliate stigma.

In contrast to findings from a previous study (49), dementia family caregivers who had regular breaks during caregiving experienced higher affiliate stigma in this study. The reason may be that caregivers with regular rest have more opportunities to socialize with the outside world, with higher engagement in interpersonal relationships. As a result, they often feel anxiety and shame owing to perceptions of being discriminated by others, which, in turn, leads to affiliate stigma. Conversely, caregivers who do not take regular breaks are more physically tired and have less contact with the outside world and are thus less likely to feel judged by others. This may explain their lower level of affiliate stigma relative to caregivers who had regular rest.

In summary, this study examined dementia family caregivers’ affiliate stigma levels from the perspective of dimensions of caregiver burden. The results may provide a basis for formulating targeted intervention strategies. Notably, demographic variables, such as family caregivers’ gender, religious beliefs, marital status, monthly household income, relationship with patients, knowledge of dementia and patients’ religious beliefs, marital status, types of medical insurance, and self-care ability did not enter the regression model, although they were found to have a significant influence on affiliate stigma in the univariate analysis. The reason may be that there are correlations between these variables; therefore, their effects on affiliate stigma are counteracted in the regression model. Future studies are recommended to further explore the relationship between these demographic variables. In addition, there may be other mediating variables between these demographic variables and affiliate stigma, which must also be further explored.

## 6 Limitations

This study has some limitations. First, the cross-sectional design did not allow us to determine causality between variables. Future longitudinal studies are necessary to further explore the causal relationship between variables. Second, the data were collected using self-reported questionnaires, which might be susceptible to self-report bias. Indicators that are more objective should be used in future studies. Third, only one main variable was included in addition to the demographic variables, which may not fully explain the factors that influence affiliate stigma in the present group. Studies involving more variables are necessary in the future. Fourth, the purposive sampling method was used to recruit target participants; however, the participants were sourced from only one dementia family caregiver online communication group. Future studies should aim to recruit participants from multiple centers to improve the generalizability of the study results. Finally, the online data collection method made it impossible to control the quality of the data filling process. Future research should adopt the on-site questionnaire collection method to ensure the quality of data.

## 7 Conclusion

Dementia family caregivers showed a moderate to high level of affiliate stigma, warranting urgent attention and efforts to mitigate this burden. Dementia family caregivers who had regular breaks during patient care, higher time-dependent burden, developmental burden, and physical burden and lower social burden exhibited higher levels of affiliate stigma.

## 8 Implications for practice

Dementia family caregivers’ affiliate stigma can be reduced by regulating their breaks during patient care, time-dependent burden, developmental burden, physical burden, and social burden. Specifically, we recommend that healthcare providers: (1) help dementia family caregivers understand the characteristics of the disease and establish an appropriate attitude toward dementia so as to enable them to reduce the impact of external bias; (2) provide development advice and help identify better development opportunities for dementia family caregivers; (3) provide professional caring knowledge and skill training in regard to dementia care to reduce the harm caused by the caring process to the family caregivers’ physical health; (4) build a social support system with multiple subjects (medical staff, community workers, and social workers) collaborating to relieve affiliate stigma of the family caregivers and reduce their social burden.

## Data availability statement

The raw data supporting the conclusions of this article will be made available by the authors, without undue reservation.

## Ethics statement

The studies involving humans were approved by the Ethics Review Board of Wenzhou Medical University (NO.2022–003). The studies were conducted in accordance with the local legislation and institutional requirements. The participants provided their written informed consent to participate in this study.

## Author contributions

YS: Data curation, Investigation, Supervision, Validation, Writing – original draft, Writing – review & editing. SD: Supervision, Writing – original draft, Writing – review & editing. ZL: Data curation, Writing – original draft. MX: Investigation, Writing – original draft. HZ: Investigation, Writing – original draft. SL: Investigation, Writing – original draft. JL: Writing – original draft, Writing – review & editing.
